# Reward Sensitivity and Noise Contribute to Negative Affective Bias: A Learning Signal Detection Theory Approach in Decision-Making

**DOI:** 10.5334/cpsy.102

**Published:** 2024-05-09

**Authors:** Isabel K. Lütkenherm, Shannon M. Locke, Oliver J. Robinson

**Affiliations:** 1 Institute of Cognitive Neuroscience, University College London, UK; 2 Laboratoire des Systèmes Perceptifs, Département d’Études Cognitives, École Normale Supérieure, PSL University, CNRS, Paris, FR; 3 Clinical, Educational and Health Psychology, University College London, UK

**Keywords:** Negative Affective Bias, Signal Detection Theory, Mood Disorders, Reward Sensitivity, Reinforcement Learning, Prospect Theory

## Abstract

In patients with mood disorders, negative affective biases – systematically prioritising and interpreting information negatively – are common. A translational cognitive task testing this bias has shown that depressed patients have a reduced preference for a high reward under ambiguous decision-making conditions. The precise mechanisms underscoring this bias are, however, not yet understood. We therefore developed a set of measures to probe the underlying source of the behavioural bias by testing its relationship to a participant’s reward sensitivity, value sensitivity and reward learning rate. One-hundred-forty-eight participants completed three online behavioural tasks: the original ambiguous-cue decision-making task probing negative affective bias, a probabilistic reward learning task probing reward sensitivity and reward learning rate, and a gambling task probing value sensitivity. We modelled the learning task through a dynamic signal detection theory model and the gambling task through an expectation-maximisation prospect theory model. Reward sensitivity from the probabilistic reward task (*β* = 0.131, p = 0.024) and setting noise from the probabilistic reward task (*β* = –0.187, p = 0.028) both predicted the affective bias score in a logistic regression. Increased negative affective bias, at least on this specific task, may therefore be driven in part by a combination of reduced sensitivity to rewards and more variable responses.

## 1. Introduction

Negative affective biases, the tendency to prioritise negative over positive information, are a core feature of mood disorders (Everaert et al., 2017; Roiser et al., 2012) and are found, for instance, in emotional perceptual processes (Park et al., 2016) as well as decision-making (Aylward et al., 2020). Targeting these negativity biases is the goal of many current treatment options, but many mood disorder patients fail to recover with our current treatments. Therefore, understanding the precise mechanisms of negative affective biases is crucial to a better understanding of mood disorders and how to treat them.

To study negative affective biases, a translational decision-making paradigm has been developed. Negative affective bias on this task is measured as a decision-making bias on ambiguous trials away from high-reward responses, i.e., interpreting an ambiguous trial as having a low-reward outcome. Within this ambiguous-cue decision making paradigm, negative bias was higher in a depressed human clinical group (Aylward et al., 2020), a rodent model of depression (Hales et al., 2016) and is related to individual differences in depression scores in the general population (Daniel-Watanabe et al., 2022).

Biases can be understood as suboptimal decision-making. Using a signal detection theory (SDT) model, a reanalysis of the data of the original ambiguous-cue decision-making task testing negative affective bias in a human clinical population (Aylward et al., 2020) linked mood disorders to decision conservatism (Locke & Robinson, 2021). Within the SDT framework conservatism is a particular type of sub-optimal behaviour, where the criterion is placed close to the neutral location showing an absence of a strong preference for one choice over the other. In an unequal reward context, an increased propensity to consider the low-reward option, in effect treating unequal reward options as more equal, is both a conservative shift in criterion placement and a bias away from the higher reward i.e., a negative affective bias (Locke & Robinson, 2021).

The observed conservatism/negativity bias on the ambiguous-cue decision-making task may be driven by a host of underlying processes. Broadly speaking, optimal perceptual decision-making is influenced by the participant’s sensitivity, knowledge, and their learning of the task environment set up (stimuli probability and pay-off structures) (for a more comprehensive discussion on drivers of sub-optimal perceptual decision making see Rahnev and Denison (2018)). Locke and Robinson (2021) identified multiple possible mechanisms leading to suboptimal conservative shifts on the ambiguous-cue decision-making task: incorrect beliefs about prior probabilities and performance, undervaluing rewards, learning sub-optimally in rewarding contexts and a preference for accurate responding. In this study, we test whether sub-optimal reward-learning or diminished subjective value representations underly the original measure of negative affective bias.

To behave optimally on tasks related to reward, participants need to learn about the choice contexts in which reward is given. From a SDT perspective, sub-optimal reward learning can be tested using probabilistic reward tasks (PRT) (Locke & Robinson, 2021; Pizzagalli et al., 2008). In these tasks, correct responses are rewarded probabilistically, keeping the reward magnitude and priors equal between two response options. As the probabilistic reward associations change throughout an experiment, both a decreased reward sensitivity (i.e., an individual is less interested in a given reward) and a decreased reward learning rate (a slower behavioural update in response to rewards) should be considered when exploring reward learning as a source of sub-optimality. We, therefore, predict that a conservative shift away from the high-reward option on the ambiguous-cue decision-making task should be underscored by a reduced sensitivity to reward during learning and/or a suboptimal reward learning rate.

Diminished subjective value representations are linked to conservative responding as modelled under SDT (Ackermann & Landy, 2015; Locke & Robinson, 2021). Value sensitivity is distinct from reward sensitivity in that value sensitivity refers to the subjective evaluation of the magnitude of a potential future reward outcome, whereas reward sensitivity refers to how received reward outcomes shape subsequent behaviour and beliefs. When maximising reward in option-valuation tasks, the criterion placement shift depends on maximising the expected gain of subjective reward values. When the expected gain of values does not differ between high and low objective reward values, in effect the two unequal reward options are treated as equal, the criterion is shifted towards the neutral position and results in a conservative behavioural response. Therefore, we expect diminished subjective value representations of rewards to lead to more negative affective bias on the ambiguous-cue decision-making task.

In sum, this paper conceptualises negative affective bias as a conservative criterion shift and builds on existing hypotheses about the origins of task conservatism to tease apart the mechanisms that contribute to negative affective bias. Previous reward decision-making accounts on conservatism stress the importance of considering static and dynamic reward contexts when studying the origin of biases away from high rewards (Locke & Robinson, 2021). We therefore related multiple aspects of reward processing in our study, namely a general, subjective loss of representing the accurate value of reward magnitude and a loss of reward sensitivity in dynamic contexts, to the negative affective bias signature of the original decision-making task. We replicated the online version of the original ambiguous-cue decision-making task (Daniel-Watanabe et al., 2022) to obtain the original negative affective bias measure. We adapted Norton et al. (2019) task to a probabilistic reward learning task to probe the relationship between our negative affective bias signature, reward sensitivity and learning rate. We chose to adapt this design, as it allowed us to model conservative reward processing and learning rate separately in a dynamic SDT model. Further, dynamic SDT models can unify learning mechanisms with SDT decision-making and therein model a respondents’ degree of conservatism in a dynamic learning context. We predicted that conservative behaviour on the learning task due to diminished sensitivity to reward would be associated with more negative affective bias. Participants also completed a gambling task, and we modelled their risk aversion, loss aversion and inverse temperature parameter through a Prospect Theory model. We predicted that diminished representations of the subjective value of rewards, as modelled by our risk aversion parameter, would be associated with more negative affective bias on the ambiguous-cue decision-making task.

## 2. Methods

### 2.1 Participants

We recruited 150 participants (98 women, mean age 39 years) online through Prolific (www.prolific.co) using the following selection criteria: 18 years or older, spoke English fluently, and had no cognitive impairment, neurological impairment, or prior head injuries. The data of two participants was not complete, leaving a final sample of 148 participants who completed all three tasks. A sample size of 105 participants is required to achieve 90% power in a two-tailed correlation (at *p* = 0.05, for effect size d = 0.3 for the correlation between two tasks). We increased our sample size as the difficulty of the dynamic SDT task was predicted to have high sensitivity analysis exclusion rates based on pilot data. The study was approved by the University College London Research Ethics Committee (protocol number: 15253/001). All participants received details of the experimental procedures and provided consent via an online form prior to participation in the experiment. Participants were reimbursed at a rate of £7.50/hour with the chance to win a £1 bonus depending on task performance.

### 2.2 Experimental Design

The experiment was conducted on the Gorilla platform (www.gorilla.sc) where each participant completed three online, computerised tasks: 1) a dynamic reward task, in which reward is given probabilistically, 2) a replication of the original ambiguous-cue decision-making task, with unequal reward-category associations, and 3) a classic gambling task, with mixed-gamble and gain-gamble payoff structures, in this order. Participants completed the dynamic reward task first, as pilot testing revealed a risk of non-engagement with this task. Participants failing comprehension (N = 88) and attention checks (N = 2) for the dynamic reward task were redirected out of the study and were not part of our final N = 148. Each experimental session lasted approximately 45 minutes.

### 2.3 Affective Bias Task: the Ambiguous-Cue Decision-Making Task

Adapting the experimental paradigm of Daniel-Watanabe et al. (2022), participants completed an ambiguous-cue decision-making task consisting of 20 learning trials and 120 test trials.

**Figure 1:**
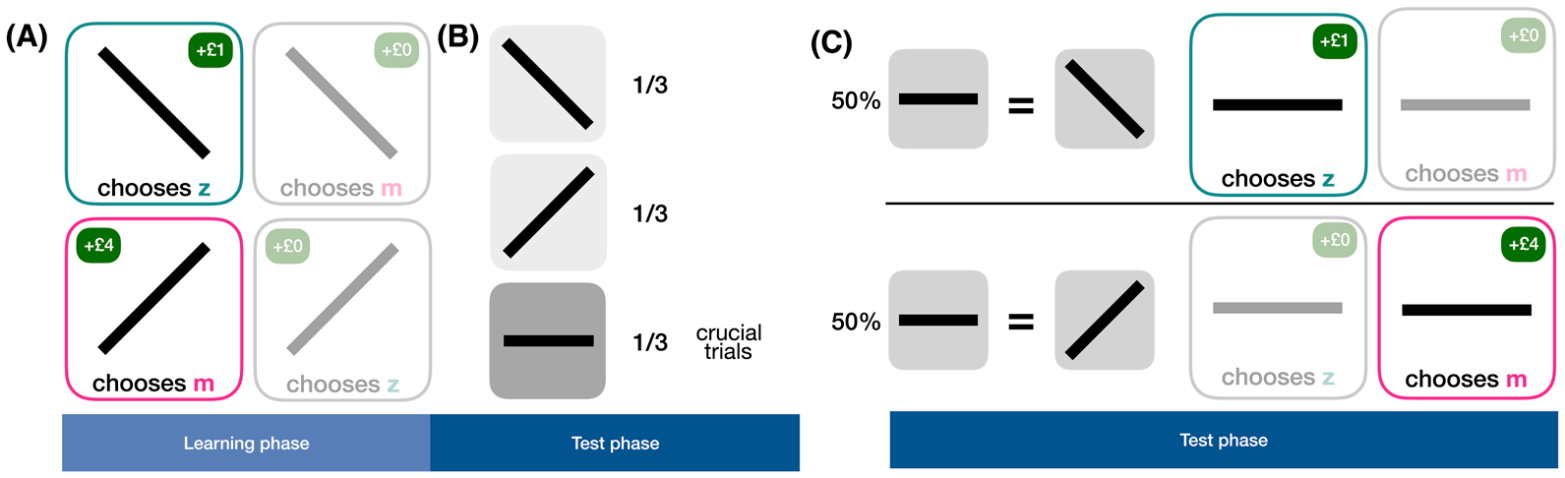
Schematic representation of the affective bias task set up and scoring procedure. **A)** In the learning phase (20 trials), participants learned associations between stimuli (left-and right-tilted lines), responses (“z” or “m” respectively) and reward outcomes (counter-balanced across participants) through trial-and-error. Here we depicted one counterbalanced reward-contingency condition. Participants who pressed “z” when seeing a left-slanted line received 1 virtual pound. When seeing a right-slanted line, an “m” response received 4 virtual pounds. **B)** The testing phase consisted of three equally likely appearing stimuli: the left-slanted line, the right-slanted line, and a horizontal line. The horizontal line is called “ambiguous stimulus” due to its intermediate representation and was used to calculate the affective bias score. **C)** A schematic representation of ambiguous trials and rewards. On half of the trials, the ambiguous stimulus belonged to the left-tilt category, and on the other half to the right-tilt category, unbeknownst to the participant. If participants responded with the correct category-response association, they received the reward associated with that category.

In the learning phase, participants saw a thick black line tilted either 45° left or right of vertical. The line was presented for 1000 ms, followed by a fixation cross of 750 ms. Participants were instructed to respond with “z” or “m” and learnt the correct association for reward (left-tilt requiring a “z” response and right-tilt a “m” response) through trial and error. Upon a correct response, positive feedback was given for 750 ms (“Correct, win £4” or “Correct, win £1”). Incorrect or late trials incurred an experimental pause of 3250 ms with feedback reading “incorrect!” or “too late, timeout!” before proceeding to the subsequent trial. Both stimuli were equally likely to be presented, but their reward structure was unequal: counterbalancing the stimulus-reward associations, one stimulus-response pair was awarded a hypothetical, virtual £1, and the other hypothetical £4 (Figure 1A).

In the testing phase, participants saw the same task setup as in the learning phase. However, an intermediate stimulus, a horizontal line (0°), was added. The three potential stimuli were again presented equally often (i.e., 33% of trials). Unbeknownst to the participant, the horizontal stimulus of a given trial randomly belonged to either the “left-tilt” or “right-tilt” category. If the correct response was given according to this random assignment, the reward on those trials mirrored the response-reward associations of the randomly allocated stimulus category (Figure 1C).

As per Daniel-Watanabe et al. (2022), we measured negative bias as the proportion of high reward choices on ambiguous stimuli. That is, we quantified how often an individual participant selected the response associated with the £4 rewarded category when they saw the horizontal stimulus and did not time-out, giving us the dependent variable p(mid as high). Given the task contingencies, p(mid as high) = 1 is the ideal strategy to maximise rewards. Random responding would correspond to p(mid as high) = 0.5. Negative affective bias is inversely proportional to p(mid as high).

### 2.4 Dynamic Reward Task: the Learning Task

**Figure 2:**
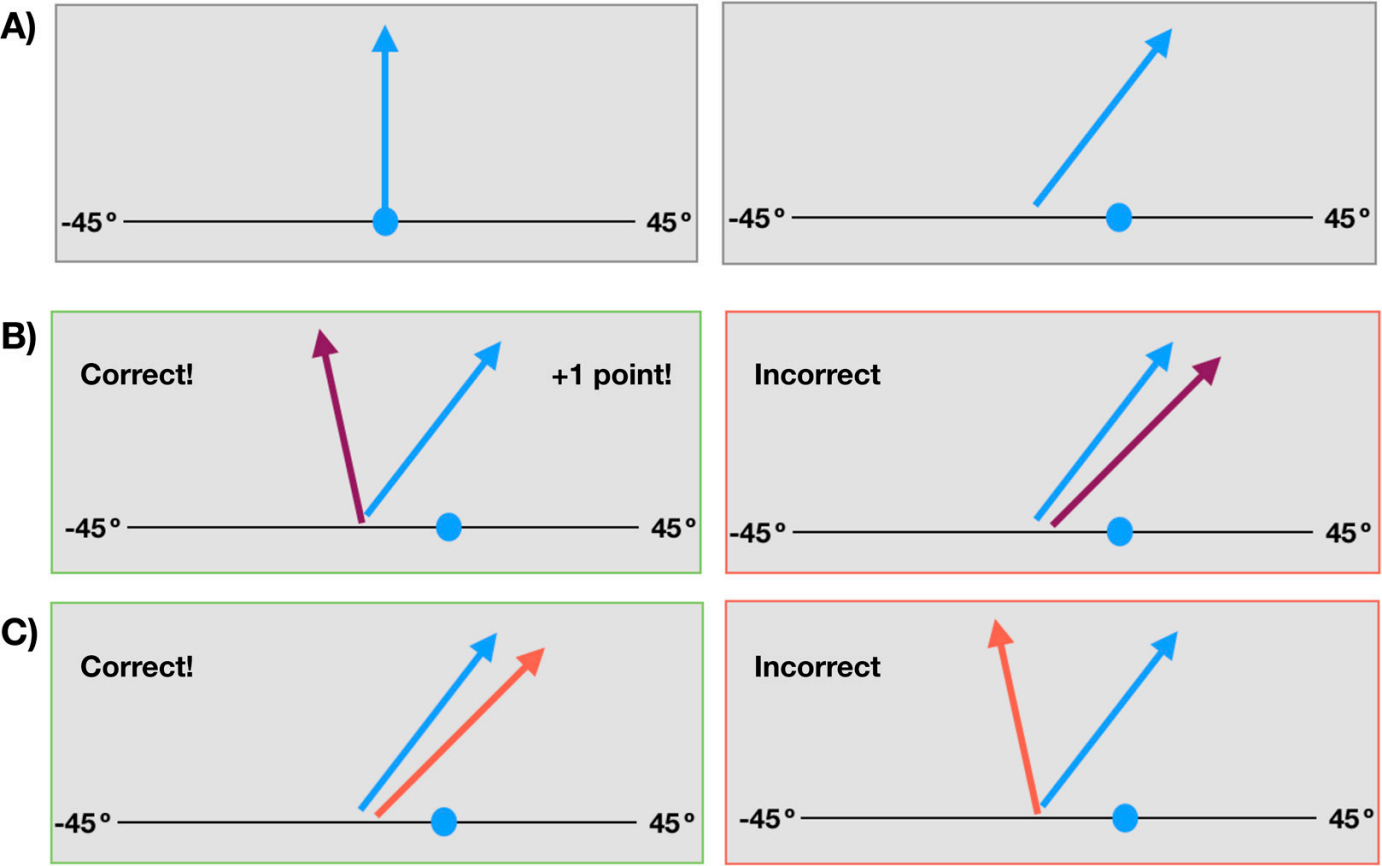
A schematic of the scoring of correctness of the dynamic reward task. A) Participants moved a blue arrow using a slider. This corresponded to their task response. Once they let go of the slider, a second arrow was superimposed. B) On half of the trials, the second arrow is from category purple (P). The tip of the purple arrow must fall left of the blue arrow tip to be scored as correct. C) On trials with superimposed category orange (O) arrows, the tip of the orange arrow must fall right of the blue tip to be scored as correct. Not all correct trials are rewarded. Instead, category reward was yoked and changed throughout the experiment, necessitating a continuous adjusting of response bias to gain maximal rewards.

The purpose of this task was to characterise how reward affects participants’ behaviour in a changing environment. To achieve this, we adapted Norton et al. (2019)’s overt criterion placement task designed to study perceptual decision-making in an environment with dynamic rewards. On each trial, participants saw a blue arrow on the screen with a randomised starting position. Participants were instructed to move the arrow between –45 and 45 degrees of vertical via an onscreen slider using their mouse or trackpad, with the final position of the slider recorded as the behavioural response. After letting go of the slider, a second coloured arrow was superimposed (Figure 2). With equal probability, this arrow was either purple (category P), or orange (category O). The orientation of this second arrow was sampled from the corresponding Gaussian distribution for that category (purple µ = –15°, orange µ = 15°, σ = 20°). Sampling was conducted for each trial independently. Correct categorisation was given the feedback “Correct”, and incorrect categorisation “Incorrect”, for 200 ms before the start of the next trial. Trials were counted as correctly categorised if the orange arrow was titled more rightward than blue arrow, or the purple arrow was tilted more leftward of the blue arrow. On some trials, correct categorisation would lead to a virtual reward. Such trials additionally displayed the feedback “+1 point!” and a point was added to a tally of the participant’s total score. After the first 5 trials and then every 9 trials, participants are asked the question: “Which arrow is currently rewarded more often?” and they responded either “purple” or “orange”.

Participants were randomised to six pseudo-randomised reward-contingency structures, which ensured that the order was counterbalanced with regard to the reward probabilities of the colour categories. Each reward-contingency structure had a total of 194 trials, divided into 5 reward-contingency epochs, each lasting between 38–45 trials. Epochs were defined by yoked category-reward contingency ratios (50:50%, 20:80%, 80:20%, 35:65%, 65:35%). The first epoch always rewarded both categories (orange and purple) equally. Thereafter, reward contingencies of the two categories were unpredictable and unequal but yoked. This reward structure requires the participant to adjust their response bias (i.e., the orientation of the blue arrow) differently across time to maximise rewards.

For each trial, we recorded the participant’s slider end orientation position as their response, which divided the decision space into “orange category” and “purple category” choices. Combined with the random sample from the category distribution, this determined the correctness of their choice. We used this information in our SDT modelling as our trial-by-trial empirical criterion placement (*c*) (see 2.4.1).

#### 2.4.1 Modelling for the dynamic reward task

To model the dynamic reward task, in which participants had to continuously learn reward changes and map their decisions, we fit a dynamic signal detection theory model with four free parameters. We computed a gain parameter G, (–∞ ≤ G ≤ ∞), interpreted as reward sensitivity, with 1 < G < ∞ corresponding to liberal and 0 < G < 1 corresponding to conservative responding. That is, a liberal participant would have a disproportionately strong response to reward, whereas a conservative participant would have a diminished sensitivity to reward and instead bias their responses towards a prior which treats rewards as equal. Note that –1 < G < 0 reflects incorrect mapping of conservatism and – ∞ < G < –1 reflects incorrect mapping of liberal responses. We also computed the learning rate, α (0 ≤ α ≤ 1) of the participant interpreted as a participant’s sensitivity to new information, with higher α implying greater sensitivity; a bias parameter, b (–∞ ≤ b ≤ ∞), capturing general shifts in sub-optimal responding; and a setting noise parameter (σ) reflecting the accuracy of mapping the decision to an actual response, with larger numbers corresponding to greater inaccuracies.

A dynamic SDT model differs from a static SDT model, in that it has a standard signal detection theory component and a learning component. The learning component step of our model describes how the belief about reward probabilities is formed over time given reward feedback. We here used a learning process that was adapted from a similar dynamic decision-making task (Norton et al., 2019):


\[
{P_{O(t)}} = \alpha *{R_{(t - 1)}} + {(1 - \alpha )^{c(t - 1)}}*{P_{O(t - 1)}}.
\]

Where *P_O(t)_* is the estimation of the reward probability for the orange category on the current trial, *R_(t–1)_* is the reward value in the previous trial, *c_(t–1)_* is the correctness of the previous response (i.e., either 1 if the stimulus was correctly categorised, or 0 otherwise), α is the fitted learning rate parameter, and *P_O(t–1)_* is the estimation of the reward of the orange category on the previous trial. Note that this formulation only updates the reward-probability beliefs following correct trials, as no information about reward probability can follow an incorrect trial.

The standard SDT modelling step describes how a participant’s belief e.g., about a reward, is translated into a criterion placement i.e., a decision-making threshold (Locke & Robinson, 2021; Macmillan & Creelman, 1991). In our model we considered how the criterion placement of the observer (c), deviated from the optimal, by fitting a magnitude scaling parameter (G) and a shifting parameter (b) to the criterion placement of the optimal observer with identical reward-probability beliefs:


\[
c = G*{c_{opt}} + b.
\]

To model the actual response, we used a likelihood function given the model predictions and the setting noise(σ):


\[
{c_{response}}\sim N(c,\,\sigma ).
\]

More details about the modelling can be found in the supplementary file 1.

### 2.5 Gambling Task: the Prospect Theory Task

**Figure 3:**
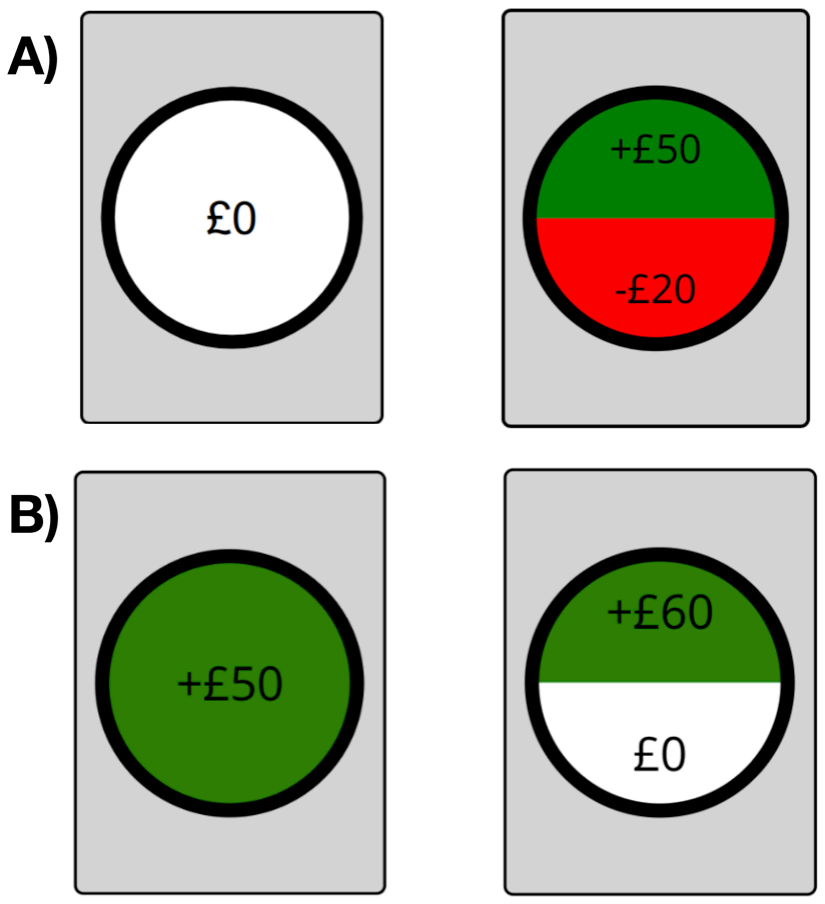
An example of a mixed-gamble and a gain-gamble trial. For 100 trials, participants made a choice between two cards, showing a safe bet (left option) or a 50/50 gamble (right option). They could be presented with either with a mixed-gamble (A) or a gain-gamble (B). Each safe bet, gamble combination was shown twice. Participants did not receive feedback. A) When safe bets gave 0 points, participants could either gain or lose a reward on the gamble. Here, there was a 50% chance to win £50 or loose £20. B) When safe bets gave a reward, the gamble consisted of winning nothing 50% of the time or winning a reward 50% of the time, here £60.

In this task, participants chose between a card showing a safe choice, or a 50/50 gamble (Figure 3) by either clicking on the image or using the left and right arrow buttons on their keyboards. Participants received no feedback on their choices. There were 100 trials in total, with an equal number of mixed-gambles (Figure 3A), where the safe bet was 0 and the gamble could incur a gain or a loss, and gain-gamble trials (Figure 3B), in which the safe bet was a gain, and the gamble either gave no gain but also no loss or a gain, which was potentially higher than the gain from the safe bet. The gamble pay-off structures can be found in the supplementary file 2. The presentation order was randomised. We used a prospect theory model to extract three parameters per participant from their gamble choices: risk aversion (ρ), loss aversion (δ), and inverse temperature (τ) (Kahneman & Tversky, 1979). For details regarding the parameter values and the model fitting procedure see the supplementary file 1 and Huys et al. (2011).

### 2.6 Statistical Analysis

To understand the relationship between our measure of negative affective bias, p(mid as high), and reward and value processing in dynamic and static contexts, a logistic regression was run in Python (version 2.7). We used all parameters obtained through the modelling of the tasks, as well as age and sex as predictors (total nine predictors) for the affective bias score. The natural logarithm of |G| was taken to make the regression more interpretable. Thereafter, G = |0.5| and G = |2|, a doubling or halving of the response gain respectively, were equally spaced. Two participants’ data was not included, due to task non-completion, resulting in a final sample size of 148. All parameters included in the regression except for sex were normalised, to facilitate effect-size comparison.

Many participants had a high noise parameter. This could indicate a high choice variability or misunderstanding of the task. Therefore, to increase the interpretability of our noise result and to reduce noise in our data more generally, we performed a logistic regression in a strict, reduced sample (N = 45). To ensure we only included the data of participants who were explicitly following the reward probability changes, we excluded participants whose accuracy score was less than 60% on meaningful probes explicitly asking for the more rewarded category. Probes did not count as meaningful if they were asked in the first epoch when orange and purple trials were rewarded equally, and after <5 trials of a probability-ratio change. To ensure a good model fit, we excluded participants with σ > = 17 and a positive log posterior. To increase the trustworthiness of the affective bias score, we excluded participants who timed out on > 10% of unambiguous trials and had an accuracy score of less than 70% of correct responses on unambiguous trials in the affective bias task.

### 2.7 Preregistration and Data Availability

The data, pre-registration and learning model code for this study can be found at https://osf.io/ewn23/.

## 3. Results

### 3.1 Descriptive Statistics

The descriptive statistics of the affective bias score and the predictors of the logistic regression can be found in Table 1.

**Table 1:** The descriptive statistics of the affective bias score and the nine parameters used in the logistic regression model to predict the affective bias score. The outcome of the logistic regression, p(mid as high), represents the affective bias sore. The demographic predictor variables included were age and sex. The predictor variables from the dynamic SDT model were the learning rate (α), the reward sensitivity (i.e., gain, ln|G|), the bias parameter (b), and the setting noise parameter (σ). The predictor variables from the prospect theory model were the subjective value of reward (i.e., risk aversion, ρ), loss aversion (δ) and inverse temperature (τ).


VARIABLE	VARIABLE SYMBOL	TASK	MEAN	SE	MEDIAN	IQR	MINIMUM	MAXIMUM

Negative Affective Bias Measure	p (mid as high)	Affective Bias Task	0.57	0.02	0.55	0.25	0	1

Age	N/A	N/A	39.23	0.99	38	17	19	78

Sex	N/A	N/A	0.66	N/A	N/A	N/A	N/A	N/A

Learning Rate	α	Dynamic Reward Task	0.23	0.01	0.23	0.17	0	0.66

Reward Sensitivity i.e., Gain	ln(|G|)	Dynamic Reward Task	–1.29	0.11	–1.27	1.58	–5.99	1.29

Bias	b	Dynamic Reward Task	–0.09	0.5	0.09	4.05	–31.63	32.73

Setting Noise	σ	Dynamic Reward Task	14.26	0.44	15.42	9.62	0.42	19.96

Subjective Value Sensitivity i.e., Risk Aversion	ρ	Gambling Task	0.68	0.02	0.69	0.41	0.22	1.3

Loss Aversion	δ	Gambling Task	2.31	0.1	2	1.57	0.4	7.27

Inverse Temperature	τ	Gambling Task	0.62	0.1	0.11	0.43	0	7.21


### 3.2 Affective Bias Measure

**Figure 4:**
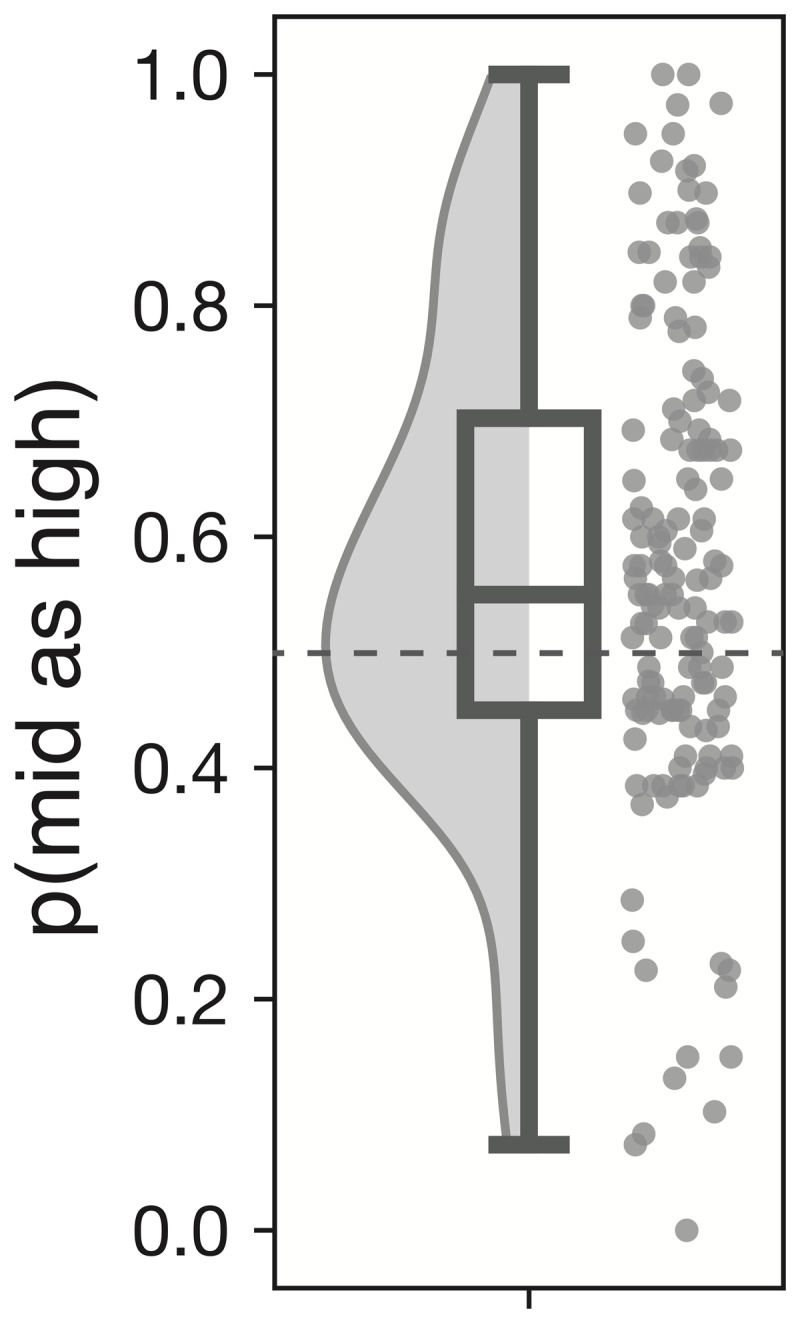
Box plot of the affective bias score with a density distribution plot and individual data points. The plot represents the affective bias score data i.e., p(mid as high) data of the full sample of 148 participants.

The median p(mid as high) of 148 participants is 0.55 (0.45–0.7). The minimum value is 0 and the maximum value is 1. We thus see that most participants behave sub optimally (i.e., have p(mid as high) <1) (Figure 4).

### 3.3 Logistic Regression

#### 3.3.1 Full sample (N = 148)

**Figure 5:**
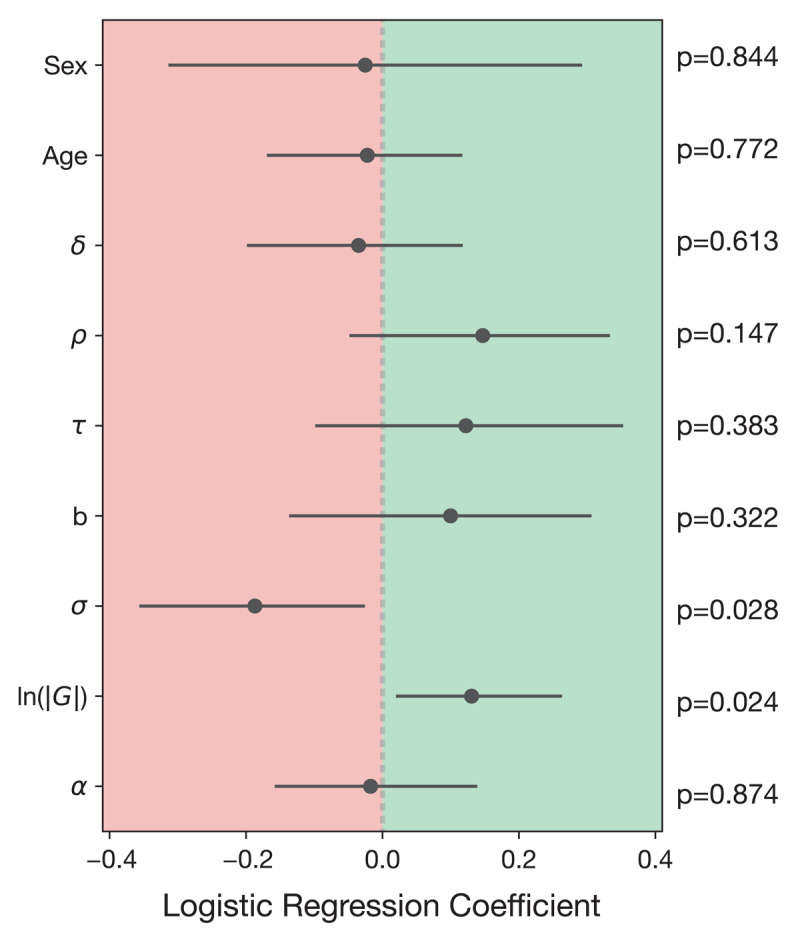
The logistic regression predicting p(mid as high) performed on the full sample (N = 148). The predictor variables were sex, age, δ = loss aversion, ρ = risk aversion conceptualising subjective value sensitivity, τ = inverse temperature, b = bias, σ = setting noise, ln(|G|) = gain parameter conceptualising reward sensitivity, α = learning rate.

P(mid as high) was significantly predicted by σ (setting noise, β = –0.187, p = 0.028) and ln(|G|) (reward sensitivity i.e., gain, β = 0.131, p = 0.024) only (Figure 5). The relationship between reward sensitivity and the affective bias score is in line with predictions; when participants have a low reward sensitivity, their p(mid as high) is lower. In a sample that includes all participants, we can infer that both choice variability and conservatism bear a relationship with response biases on the affective bias task.

#### 3.3.2 Contextualising the significant reward sensitivity result

**Figure 6:**
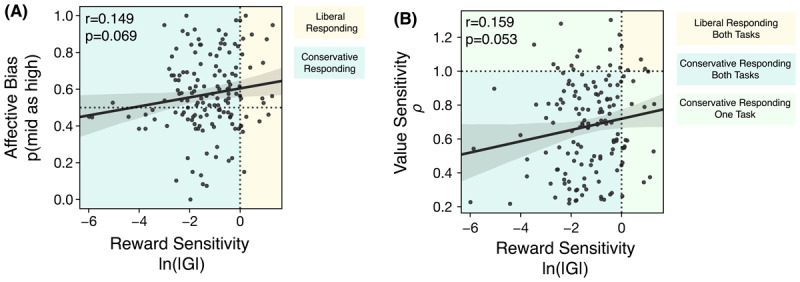
Pairwise linear correlations between reward sensitivity, affective bias and subjective value sensitivity **A)** The raw correlation between the affective bias score and reward sensitivity. There is a weak positive, non-significant relationship. The dashed horizontal line indicates no preference between the low and high reward. The dashed vertical line represents a neutral gain parameter, which neither inflates nor shrinks optimal criterion estimates. Turquoise shaded region indicates participants are conservative on the learning task, the yellow shaded region shows that participants are liberal on the learning task. **B)** The raw correlation between reward sensitivity and subjective value sensitivity. The dotted horizontal and vertical lines indicate no bias. The yellow shaded area encompasses participants who are liberal in their criterion placements and risk seeking. The upper green shaded background indicates conservatism in the gain parameter but risk seeking behaviour in the gambling task. The right hand green shaded area indicates risk aversion but inflation of reward. The turquoise shaded region indicates conservatism on both the risk aversion and gain parameter.

A simple Pearson correlation between the raw p(mid as high) and reward sensitivity parameter values (i.e., gain, ln|G|) shows a non-significant relationship; those who are less negatively biased in the affective bias task are also more sensitive to reward in the learning task (r = 0.149, p = 0.069; Figure 6A). Further, there is an insignificant trend that those who are more sensitive to reward in the learning task are also more sensitive to value in the gambling task (r = 0.159, p = 0.053; Figure 6B).

#### 3.3.3 Contextualising the significant setting noise result

We also performed a logistic regression restricted to participants who did not fail stringent data quality checks (N = 45). In this regression, p(mid as high) was significantly predicted by σ only (β = –0.436, p = 0.019) although the effect of ln(|G|) (β = 0.249, p = 0.183) was in the same direction as predicted and found in the full sample.

**Figure 7:**
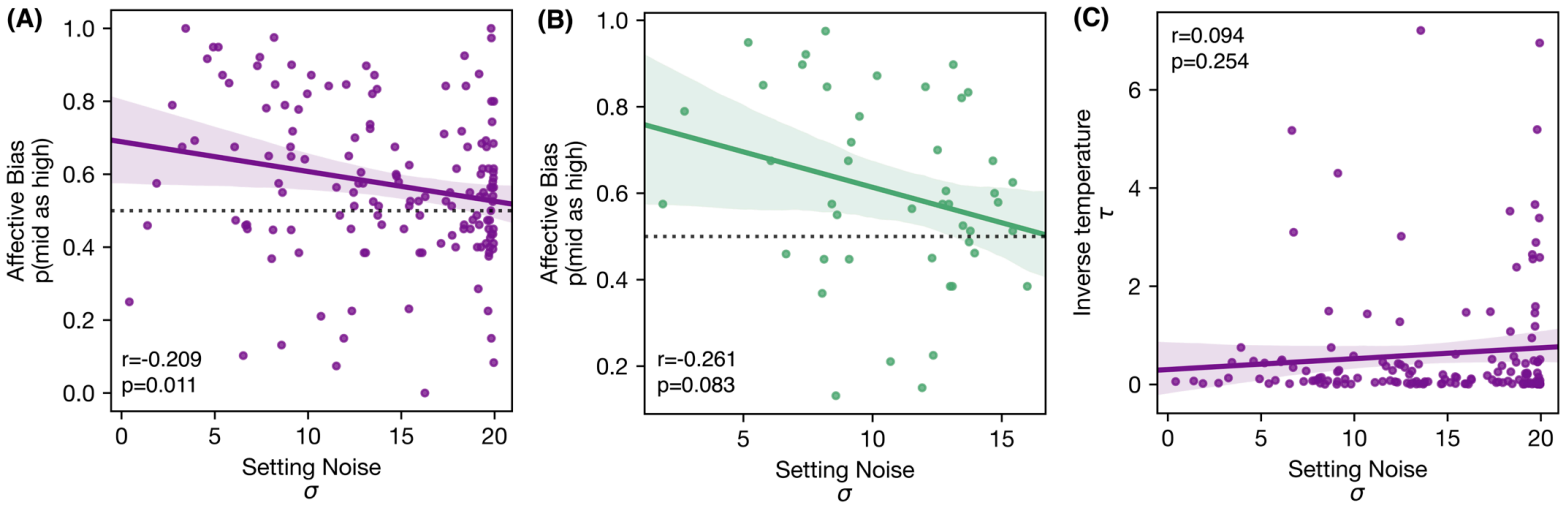
Pairwise linear correlations between setting noise, affective bias and inverse temperature **A)** Showing the raw correlation between setting noise and the affective bias score in the full sample. **B)** Showing the raw correlation between setting noise and the affective bias score in the reduced sample **C)** showing the raw correlation between setting noise and inverse temperature in the full sample.

In line with the logistic regression analysis, participants with a higher noise parameter had a lower affective bias score (i.e., they were more negatively biased) on a Pearson correlation between σ and p(mid as high) (full sample: r = –0.209, p = 0.011; reduced sample: r = –0.261, p = 0.083; Figure 7A–B). The significant correlation of the full sample shows that most participants with the highest noise parameters respond conservatively on the affective bias task (p(mid as high) around 0.5), instead of with a true negative bias preferring the low reward option (p(mid as high) < 0.5). This might indicate that people who are noisier on the learning task, are also more inconsistent in their responses on the horizontal trials of the ambiguous-cue decision-making task.

Interestingly, setting noise from the learning task and inverse temperature from the gambling task are not correlated (r = 0.094, p = 0.254; Figure 7C). This relationship holds when transforming inverse temperature into a log space and across the sensitivity exclusion. Thus, we show no relationship between setting noise and inverse temperature even though they both represent inconsistent task-responding, indicating that these two parameters correspond to might different noise sources.

## 4. Discussion

In the present study, we investigated reward sensitivity, subjective value sensitivity, learning rate and noise as possible mechanisms underscoring a replicated measure of negative affective bias, which previously revealed that people with depression choose high-reward responses less frequently than healthy controls (Aylward et al., 2020; Daniel-Watanabe et al., 2022) and are more conservative in their criterion placing than healthy controls (Locke & Robinson, 2021). Our results reveal a significant association between reward sensitivity (i.e., gain, ln|G|) and affective bias; the less sensitive a participant is to reward in a probabilistic learning task, the more negatively biased they are in the ambiguous-cue decision-making task. We failed to find an association between reward learning rate and negative affective bias. We further failed to find an association between subjective value sensitivity (i.e., risk aversion, ρ) and affective bias. This indicates that reward sensitivity and not value sensitivity, that is detecting the chance of reward rather than accurately gauging the magnitude of a reward, might underscore task conservatism on our metric of negative affective bias. Additionally, we found an association between affective bias and noise. This indicates that choice variability might play a role in determining the affective bias score.

We predicted conservative choices on our affective bias task, indicative of a larger negative affective bias propensity, would be linked to a reduced sensitivity to reward during learning. We found some support for this prediction. Specifically, we found a relationship between our gain parameter in the learning task and our negative affective bias score in the full sample: when participants have a low absolute gain parameter, their probability of choosing a high reward option on ambiguous trials is lower. A smaller absolute gain parameter shrinks the criterion placement estimate and therein biases participants towards conservative responding i.e., participants behave as if the two reward probabilities are more similar than they are. As the criterion placement is dependent on a transformation of the reward-probability estimate, this indicates the participants’ sub-optimality is due to a reduced response to reward. This result is in line with the results of the to date most extensive probabilistic reward task learning set in depressed participants, which showed depressed participants had a conservative bias and specifically reduced reward sensitivity rather than abnormal learning per se (Admon & Pizzagalli, 2015; Huys et al., 2013; Pizzagalli et al., 2008).

We also predicted that a diminished subjective sensitivity to value would drive a bias away from high rewards on our affective bias task. While rewards refer to the objective value received after an outcome, value refers to an individual’s subjective evaluation of the desirability of a potential outcome, i.e., their subjective representation of the reward (Kahneman & Tversky, 1979). Contrary to our hypothesis, we did not find a positive association between subjective value sensitivity and the affective bias score. This prediction was made as a small subjective value sensitivity parameter shrinks the expected gain of subjective values and results in a conservative criterion placement shift (Ackermann & Landy, 2015; Ulehla, 1966). Further, our affective bias signature is associated with depression (Aylward et al., 2020; Daniel-Watanabe et al., 2022), and depression has been linked to undervaluing rewards in static contexts (Halahakoon et al., 2020). Therefore, in our sample, decreased subjective value sensitivity does not seem to be the source of task conservatism. This indicates a diminishment of valuing reward does not incur a weaker preference for a high-reward response association on ambiguous cues on the affective bias task. Combining this with the significant reward sensitivity effect above shows that not choosing high reward options in uncertain contexts is not related to an a priori disregard of the value of reward on the affective bias task but to a difference in experiencing the presence or absence of rewards. In our affective bias task, participants were not given explicit response instructions, but learnt reward contingencies through trial and error. Therefore, it makes conceptual sense, that a reduced sensitivity to positive feedback does not incur a bias toward high-reward response choices on this task.

In our study, setting noise also significantly predicted the affective bias score. A higher noise parameter in the dynamic reward task was related to a tendency to not choose the high reward option on ambiguous trials in the affective bias task. The setting noise parameter signifies how accurately the criterion placement decision is mapped to the actual response in the learning task. This result was robust to exclusions for excessive noisiness. When looking at the significant simple correlation between the setting noise parameter and the affective bias score, the relationship is not driven by true negative affective bias scores (p(mid as high) < 0.5) but instead by conservative responses (p(mid as high) ~0.5) (see Figure 7A–B). However, it is important to note that the setting noise parameter can be influenced by a variety of participant behaviours, attributable to task-specific dimensions (e.g., divergent exploration strategies, misunderstanding of the task) or non-specific task dimensions (e.g., executive function, intelligence, attention, concentration).

In the full sample, it is plausible the relationship between setting noise and p(mid as high) was driven by a misunderstanding of the task or a lack of concentration. This sample included participants who failed to correctly respond to questions probing explicit knowledge of the more frequently rewarded category in the learning task and further included respondents who gave incorrect answers to unambiguous trials of the affective bias task. Considering depression is associated with executive-function processing difficulties (Varghese et al., 2022), particularly attentive difficulties (Keller et al., 2019), an affective bias score of 0.5 could represent stochastic noise in a forced two-choice task. However, our stringent sample allows us to indirectly differentiate between possible sources of setting noise. Despite being underpowered, the significant association between noise and affective bias remains after the exclusion of participants whose data indicates a possible lack of concentration or task understanding. Therefore, the association is likely not just linked to random responding. Instead, an elevated setting noise parameter value could also be the result of divergent decision-making behaviour and strategies. As participants need to learn the response and reward associations (half of the ambiguous trials belong to the leftward slant category and the other half belong to the rightward slant category, rewarding correct category-response associations with the category’s reward magnitude) on the affective bias task, a conservative affective bias score might represent over-exploration or within-task strategy switching, while a higher p(mid as high) score might be representative of a more stable reward exploitation strategy. If this were true, our metric of affective bias could represent a greater tendency to explore the reward task space, instead of a reduced preference for high rewards. Thus, the affective bias score could plausibly be underscored by alternative task engagement strategy (exploration-exploitation trade-off), choice variability, or indecisiveness. This would concur with insights from the depression literature, which linked depression to more inconsistent task choices on reinforcement learning tasks (Pike & Robinson, 2022) and linked choice variability to anhedonia symptoms on a PRT (Huys et al., 2013). Given participants had to learn the response reward structure and p(mid as high) was statistically related to reward sensitivity and setting noise in our powered sample, the behavioural signature is most likely influenced by multiple processes, and not a pure measure of negative *affective* bias.

There is a need to understand mechanisms behind negative biases in order to develop more targeted pharmacological approaches and psychological treatments for mood disorders, as bottom-up negative biases seem to be implicated both in the development and the recovery of mood disorders (Godlewska & Harmer, 2021; Roiser et al., 2012). The ambiguous-cue decision-making task we used to probe negative affective bias is a simple behavioural task, which showed reliable associations with depression in a general and clinical population in humans as well as a mouse model of depression (Aylward et al., 2020; Daniel-Watanabe et al., 2022; Hales et al., 2016). The task therefore seemed promising for translational research of treatments and mechanisms. Our study in a general population sample suggests the affective bias score is driven by a multitude of processing biases and decision-making strategies and might therefore not be the cleanest approach for future translational research. Nonetheless, our result can also inform the direction of future pharmacological and non-pharmacological trials aiming to improve mood disorder treatment. Given the replicated association between our negative affective bias signature and depression (Aylward et al., 2020; Daniel-Watanabe et al., 2022; Hales et al., 2016), we suggest future research approaches target reward pathways in depression treatment. This suggested research direction would concur with evidence from a randomised clinical trial targeting reward hyposensitivity (Craske et al., 2023). Treatment focusing on positive affect was superior to treatment focusing on negative affect. Further, in the positive affect arm, the improvements on reward anticipation-motivation and responses to reward attainment correlated with improvements in positive affect, anhedonia and depression and anxiety (Craske et al., 2023).

Our study had several benefits. In SDT, conservatism is a commonly observed behaviour that deviates from the optimal strategy, where an individual places their decision criterion close to the neutral location, indicating no strong preference for either choice (Green & Swets, 1966; Ulehla, 1966). A previous study linked our metric of negative affective bias to a conservative shift in criterion placement (Locke & Robinson, 2021). To better understand latent constructs, it is important to study their relationship to other related and commonly used measures within the same sample. In our case, previous studies on responses to gains have shown individual variation in both static and dynamic contexts (Halahakoon et al., 2020; Pike & Robinson, 2022) which exemplifies the importance of precisely testing reward/value-related sub-processes which lead to biases on cognitive tasks such as the affective bias signature on the ambiguous-cue decision-making task. A strength of our approach was therefore that we were able to test two underlying constructs as possible sources of task conservatism (Locke & Robinson, 2021). Our experiment approached this by studying how reward feedback influenced the conservatism of criterion placements and through the risk aversion parameter of a gambling task. This let us tackle a conceptual difficulty in the depression literature distinguishing an individual’s response to positive feedback in the reward outcome phase, from disrupted appraisal processes of value in the decision-phase (Chung et al., 2017). Therein, we were able to test whether disrupted value representations or reward-feedback processes drove task conservatism. Collectively, our analysis indicates that most respondents were conservative in subjective value sensitivity and reward sensitivity measures (Figure 6b). Further, participants who were conservative in their option valuation were also conservative in their reward sensitivity, although the relationship did not reach statistical significance. Thus, conservatism on the affective bias task is driven not by an overall diminishment of valuing reward, but probably at least in part by experiencing the reward feedback differently.

The SDT framework is also a useful approach when studying biases, as it relates behavioural observations to distinct cognitive processes: sensitivity and bias (Green & Swets, 1966). We exploited this approach and developed a novel probabilistic reward task. This allowed us to integrate optimality into our model, which is an improvement over previous SDT modelling of probabilistic reward tasks studying biases in depression. Further, our new task is a probabilistic reward learning task during which participants need to make overt criterion decisions. This approach yields a richer dataset of explicit criterion choices. By requiring participants to indicate a criterion placement on every trial, we had a trial-by-trial continuous estimate of participants’ perceived optimal cut-off points, which we let our model estimate. An overt criterion task also increases confidence in attributing the source of response conservatism to conservatism tendencies. Meanwhile, two-forced choice covert criterion tasks can lead to behavioural conservatism due to decision-making strategies which smooth binary observer responses (e.g., probability matching) or increased task uncertainty due to implicit feedback leading to a stronger reliance on priors (Norton et al., 2019).

Our study also has several limitations. For the current study, participants were sampled from the general population, and we did not measure their psychopathology profiles. Instead, we rely on the replicated, previously shown association between mood disorders and affective biases to interpret our findings. To understand the relationship more confidently between mood disorders, our affective bias measure, and the sources of conservatism, the study should be replicated within a clinical sample and include clinical questionnaires. Further, future studies should try and understand the source of choice variability. The task we used is sensitive to depression, and depression is related to both altered hot cognition and cold cognition (Roiser & Sahakian, 2013). To understand if choice variability is driven by inattention, exploration-exploitation trade-offs or general choice inconsistency, more testing is required. To do so, the affective bias task could be viewed as a learning task, as participants are not explicitly told the response-reward structure. Future work with this task could compare groups where this was made explicit versus the current implicit set up. Additionally, the affective bias task could be modelled as a learning task in order to account for learning rate and choice consistency differences. Alternatively, tasks testing executive functioning, exploration-exploitation trade-off as well as an IQ test could be added to the task-battery. These next steps would improve our conceptual understanding of p(mid as high). Further, the affective bias task could be made perceptually harder. Perceptual ambiguity could be added by using circle area instead of line tilt as the feature discriminating dimension. This could rule out lack of attention as a driver of conservatism, as participants with very low perceptual sensitivity would likely not be paying attention. Moreover, from an SDT perspective, this would be beneficial as it would more clearly separates biases: for tasks in which perceptual sensitivity is high, the optimal criterion point is closer to neutral than for tasks in which perceptual sensitivity is lower (e.g., d’ = 1) (Locke & Robinson, 2021). Lastly, the experiment should be replicated in a lab, as this would allow randomisation of task order. Currently, the task order is kept constant with the learning task being first, as this allowed us to exclude participants who did not pass comprehension checks of the learning task logic. As a consequence, the gambling task was last. Here, the inverse temperature parameter showed that nearly all participants showed random behaviour on this task, which could be indicative of choice fatigue. This complicates the interpretation of our prospect theory parameter results, especially the lack of association between noise parameters across the models.

To conclude, understanding the mechanisms underlying negative affective bias is crucial for our understanding of mood disorder symptomatology and treatments. Our results show reward sensitivity, but not subjective value sensitivity probably drives task conservatism on this affective bias task. The present study has also shown that the affective bias score is most probably not driven by one underlying mechanism. Both reward sensitivity and noise (and hence unmodelled decision-making strategies) seem to drive the response bias on the affective bias task.

## Additional Files

The additional files for this article can be found as follows:

10.5334/cpsy.102.s1 Supplementary File 1. Modelling. Dynamic SDT and PT modelling details.

10.5334/cpsy.102.s2 Supplementary File 2. Gamble Payoff Structure and Sensitivity Sample.

10.5334/cpsy.102.s3 Supplementary File 3. Within-Model Parameter Correlations.

10.5334/cpsy.102.s4 Supplementary File 4. Regression Model Sensitivity Analyses.
