# Supplementary material for: Reward Sensitivity and Noise Contribute to Negative Affective Bias: A Learning Signal Detection Theory Approach in Decision-Making
Source: Comput Psychiatr. 2024 May 9;8(1):70–84. doi: 10.5334/cpsy.102 (PMC11104415; doi:10.5334/cpsy.102)
Supplement: Supplementary File 1. — Modelling. Dynamic SDT and PT modelling details. [file cpsy-8-1-102-s1.pdf]

Supplementary file 1: Modelling. Dynamic SDT and PT modelling details.

Dynamic Signal Detection Theory Model and Fitting Procedure:

In this task, participants aimed at collecting points, by categorising unseen stimuli drawn from one of two category distributions, setting a decision criterion (a blue arrow) which divided the choice space. Throughout the task, the reward frequency associated with each category distribution changed dynamically. To model this task, in which participants had to learn reward changes and map their decisions, we implemented a model with a standard signal detection theory component and a learning component. The standard SDT model describes how a participant's belief e.g., about a reward, is translated into a criterion placement i.e., a decision-making threshold (Macmillan, 1991). The learning component describes how the belief about reward probabilities is formed over time given reward feedback. We here used a learning process that was adapted from a similar dynamic decision-making task (Norton et al., 2019).

In the standard SDT model, the beliefs about the environment are mapped to a criterion. It has two components, sensitivity (*d'*) and criterion (c). We did not have to model *d'* as it was the same for all participants (*d'*=1.5). This is because the noise source in the task was exclusively the external noise in the sampling from the category distributions, and not due to internal perceptual noise, as the participant was never asked to view the high-contrast orange or purple line stimulus prior to making their decision. External noise was proportional to the orientation uncertainty of the sampling distributions for the purple and orange arrows.

In a probabilistic reward task with equal category probabilities and equal reward amounts that differ in reward probability based on category, the optimal criterion (c_{opt}) placement is computed as a function of the category reward ratio and the sensitivity. In our task, the reward ratio corresponded to beliefs about the reward probabilities of the orange and purple categories. As the two categories were yoked, this can be expressed as:

$$c_{opt} = \frac{ln(\frac{1 - P_o}{P_o})}{d'}$$

Where $\frac{1-P_o}{P_o}$ corresponds to the ratio of the reward probability estimates of the purple category $(1 - P_o)$ versus the orange category (P_o).

To model the dynamic learning process for the beliefs about the category reward contingencies, we chose to implement a reformulation of the best-fitting model of Norton et al. (2019), adapted to a variable reward contingency context. For each trial, an exponential-averaging learning function updates the reward-probability estimate for the orange category ($P_{O(t)}$) the following weighted average:

$$P_{O(t)} = \alpha * R_{(t-1)} + (1 - \alpha)^{c_{(t-1)}} * P_{O(t-1)}$$

Where $R_{(t-1)}$ is the information gained on the previous trial through reward, c_{t-1} is the correctness of the previous response (i.e., either 1 if the stimulus was correctly categorised, or 0 otherwise), α is the fitted learning rate parameter, and $P_{O(t-1)}$ is the estimation of the reward of the orange category on the previous trial. Note that this formulation only updates the reward-probability beliefs following correct trials, as no information about reward probability can follow an incorrect trial.

The SDT model of criterion placements is a normative behavioural model. However, participants rarely behave optimally. Therefore, we considered how far the placement of the slider, the criterion placement of the observer (c), deviated from the optimal, by fitting a magnitude scaling parameter (G) and a shifting parameter (b) to the criterion placement of the optimal observer with identical reward-probability beliefs:

$$c = G * c_{opt} + b$$

The gain parameter represented amplified $G[1, \infty]$ or conservative $G[0,1]$ scaling of the optimal criterion placement and therein was a measure of reward sensitivity. In cases in which G was negative, the behavioural mapping was incorrect, but the response ranges were still informative for reward sensitivities. The parameter b captured response biases such as a general category preference for either the purple or orange category. To model the actual response, we used a likelihood function given the model predictions and the setting noise (σ):

$$c_{response} \sim N(c, \sigma) \,.$$

The dynamic SDT model was fit using custom-coded RStan scripts (version 2.21.0, (Stan-Development-Team, 2022) in RStudio (version 1.3.1093). We used four Markov-Chain Monte Carlo chains per participant, each containing 10000 parameter-space samples. The first 5000 samples from each chain were discarded as warm-up samples, and the mean across chains was calculated for each parameter. We constrained our sampling to the following parameter-space: α= [0,1], G = [-5,5], b = [-40,40], σ = [0,20]. We used the following informative priors: α ~ Beta(1.3, 1.3), G ~ N(1,3), b ~ N (0, 20), σ ~ log N(1.5, 0.5).

Prospect Theory Model and Fitting Procedure:

We fit a prospect theory model with three parameters (ρ,δ,τ) on the data of our gambling task. Our subjective value sensitivity parameter ρ could take values between 0-∞ with ρ = [0,1] corresponding to being risk averse and preferring certainty over uncertainty, even if the objective payoff of the certain choice is lower, ρ = 1, being risk neutral, and $\rho > 1$ corresponding to risk-seeking behaviour, valuing uncertainty over certainty. Our loss aversion parameter δ [0, ∞] corresponded to weighing potential losses stronger than potential gains with larger δ corresponding to larger aversion to losses. The inverse temperature parameter τ [0, ∞] indicated how deterministic participants were in their choice strategy, with larger τ corresponding to more consistent choice behaviour.

We fit the model hierarchically in python (version 3.7.13), using a previously adopted expectation-maximisation algorithm as described in Huys et al. (2011), to simultaneously estimate both individual-level parameters and gaussian-distributed group-level priors. In short, we randomly initialised all values to be estimated on the E-step and found the best fitting individual parameters, given our current prior estimate. On the M-step, we updated our group-level prior parameters given the current estimate of individual parameters. We repeated these steps until the model converged. Hierarchical modelling

resulted in the individual parameters estimates for risk preference, loss preference and temperature being biased towards the mean of the group-level distributions.

Huys, Q. J. M., Cools, R., Gölzer, M., Friedel, E., Heinz, A., Dolan, R. J., & Dayan, P. (2011). Disentangling the Roles of Approach, Activation and Valence in Instrumental and Pavlovian Responding. *PLOS Computational Biology*, *7*(4), e1002028. https://doi.org/10.1371/journal.pcbi.1002028

Macmillan, N. A. C. C. D. (1991). *Detection theory : a user's guide*. Cambridge University Press.

Norton, E. H., Acerbi, L., Ma, W. J., & Landy, M. S. (2019). Human online adaptation to changes in prior probability. *PLOS Computational Biology*, *15*(7), e1006681. https://doi.org/10.1371/journal.pcbi.1006681

Stan-Development-Team. (2022). *RStan: the R interface to Stan*. In https://mc-stan.org/.
