# Supplementary material for: Reward Sensitivity and Noise Contribute to Negative Affective Bias: A Learning Signal Detection Theory Approach in Decision-Making
Source: Comput Psychiatr. 2024 May 9;8(1):70–84. doi: 10.5334/cpsy.102 (PMC11104415; doi:10.5334/cpsy.102)
Supplement: Supplementary File 2. — Gamble Payoff Structure and Sensitivity Sample. [file cpsy-8-1-102-s2.pdf]

Supplementary file 2: Gamble Payoff Structure and Sensitivity Sample.

Gamble Task Pay-off Structure:

There were 100 gambles in total, with an equal number of mixed-gamble and gain-gamble trials, with the gamble pay-off structures being depicted in Table 1 and 2 respectively. Each gamble pay-off structure was shown twice to participants.

Mixed-gamble Trials

£ 70 / £ -10 vs. £ 0	£ 70 / £ -20 vs. £ 0	£ 70 / £ -30 vs. £ 0	£ 70 / £ -40 vs. £ 0	£ 70 / £ -50 vs. £ 0
£ 60 / £ -10 vs. £ 0	£ 60 / £ -20 vs. £ 0	£ 60 / £ -30 vs. £ 0	£ 60 / £ -40 vs. £ 0	£ 60 / £ -50 vs. £ 0
£ 50 / £ -10 vs. £ 0	£ 50 / £ -20 vs. £ 0	£ 50 / £ -30 vs. £ 0	£ 50 / £ -40 vs. £ 0	£ 50 / £ -50 vs. £ 0
£ 40 / £ -10 vs. £ 0	£ 40 / £ -20 vs. £ 0	£ 40 / £ -30 vs. £ 0	£ 40 / £ -40 vs. £ 0	£ 40 / £ -50 vs. £ 0
£ 30 / £ -10 vs. £ 0	£ 30 / £ -20 vs. £ 0	£ 30 / £ -30 vs. £ 0	£ 30 / £ -40 vs. £ 0	£ 30 / £ -50 vs. £ 0

Table 1: Depiction of the gamble-safe bet trade-offs on mixed gamble trials. On mixed-gamble trials, each gamble is paired with a safe bet of £0. This means participants could either choose a 50/50 gamble on which they could win a reward or incur a loss or win nothing and therein not incur a loss by choosing the safe option 0 £.

Safe-gamble Trials

£ 100/ £ 0 vs. £ 10	£ 100/ £ 0 vs. £ 20	£ 100/ £ 0 vs. £ 30	£ 100/ £ 0 vs. £ 40	£ 100/ £ 0 vs. £ 50
£ 80/ £ 0 vs. £ 10	£ 80/ £ 0 vs. £ 20	£ 80/ £ 0 vs. £ 30	£ 80/ £ 0 vs. £ 40	£ 80/ £ 0 vs. £ 50
£ 60/ £ 0 vs. £ 10	£ 60/ £ 0 vs. £ 20	£ 60/ £ 0 vs. £ 30	£ 60/ £ 0 vs. £ 40	£ 60/ £ 0 vs. £ 50
£ 40/ £ 0 vs. £ 10	£ 40/ £ 0 vs. £ 20	£ 40/ £ 0 vs. £ 30	£ 40/ £ 0 vs. £ 40	£ 40/ £ 0 vs. £ 50
£ 20/ £ 0 vs. £ 10	£ 20/ £ 0 vs. £ 20	£ 20/ £ 0 vs. £ 30	£ 20/ £ 0 vs. £ 40	£ 20/ £ 0 vs. £ 50

Table 1: Depiction of the gamble-safe bet trade-offs on gain gamble trials. On gain-gamble trials, each gamble is paired with a safe bet that is a reward. This means participants could either win a reward by choosing the safe option or choose a 50/50 gamble on which they could win a reward that sometimes was larger than the safe bet or win nothing.

Sensitivity Sample:

A sensitivity analysis was performed with a stringently reduced sample (N=45), for which we excluded participants due to the following reasons. To ensure a good model fit, we excluded participants with $\sigma >= 17$ and a positive log posterior. To ensure we only included the data of participants who correctly followed the probabilistic reward changes in the learning task, we excluded participants whose accuracy score on the question "which category is currently more rewarded" was less than 60 % for meaningful probes. Probes did not count as meaningful if they were asked in the first epoch when orange and purple trials were rewarded equally, and after <5 trials of a probability-ratio change. To ensure the trustworthiness of the affective bias score, we excluded participants who timed out on > 10 % of unambiguous trials and had an accuracy score of less than 70 % of correct responses on unambiguous trials in the affective bias task.
