# Supplementary material for: Reward Sensitivity and Noise Contribute to Negative Affective Bias: A Learning Signal Detection Theory Approach in Decision-Making
Source: Comput Psychiatr. 2024 May 9;8(1):70–84. doi: 10.5334/cpsy.102 (PMC11104415; doi:10.5334/cpsy.102)

Supplementary File 3: Within-Model Parameter Correlations

In the full sample, the learning rate parameter and the reward sensitivity parameter are significantly correlated with a moderate effect size ($r=-0.45$, $p < 0.001$). The lower the learning rate, the more liberal the gain parameter i.e., the more reward sensitive. The moderate correlation indicates that the parameters likely represent similar constructs but are still meaningful parameters to use in our regression, as they represent enough variance independently to predict the affective bias score.

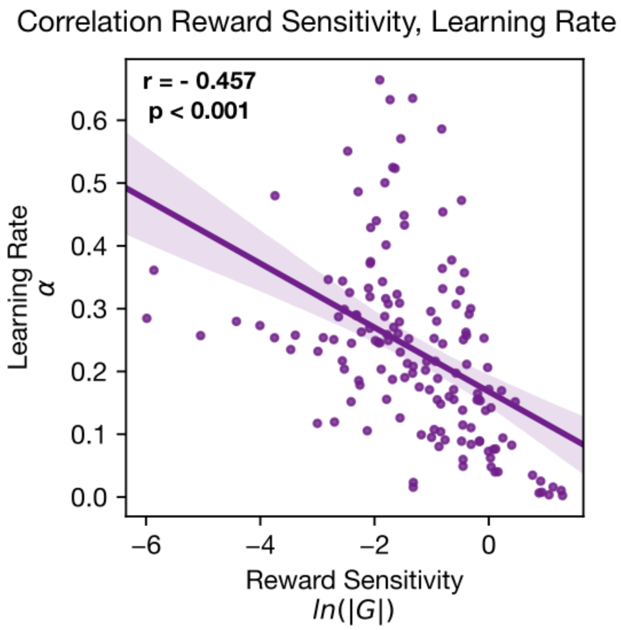

We plot all the remaining within model parameter correlations below.

Learning model parameter correlations:

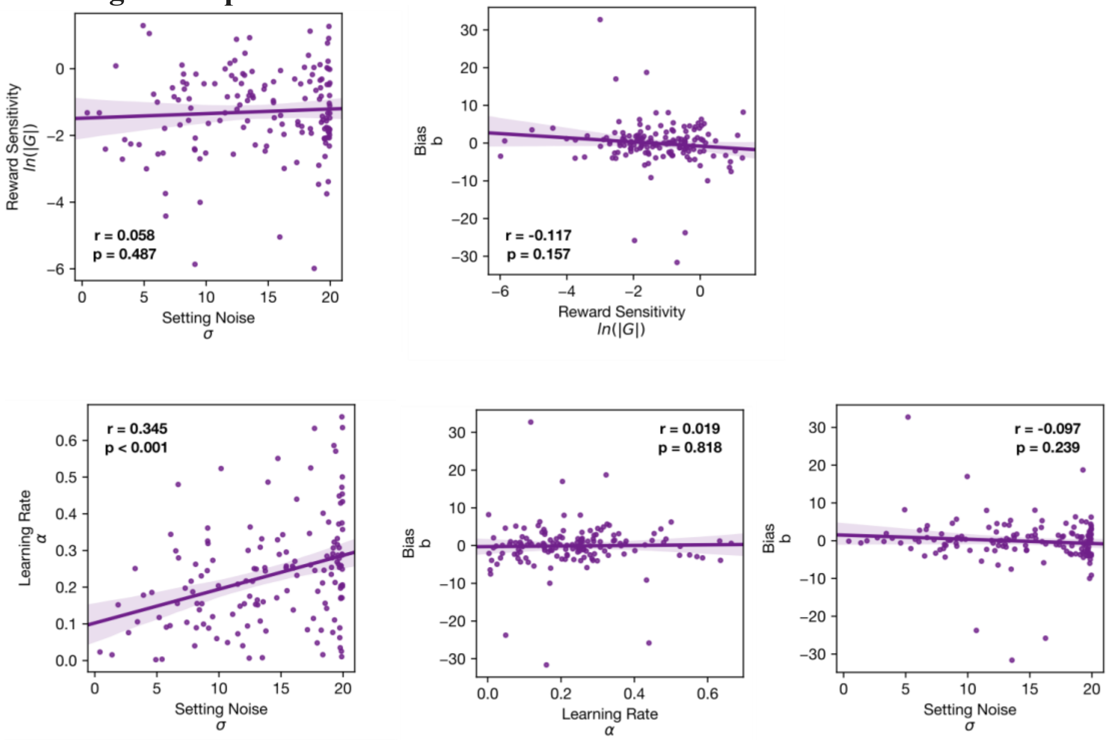

Prospect Theory Parameter Correlations:

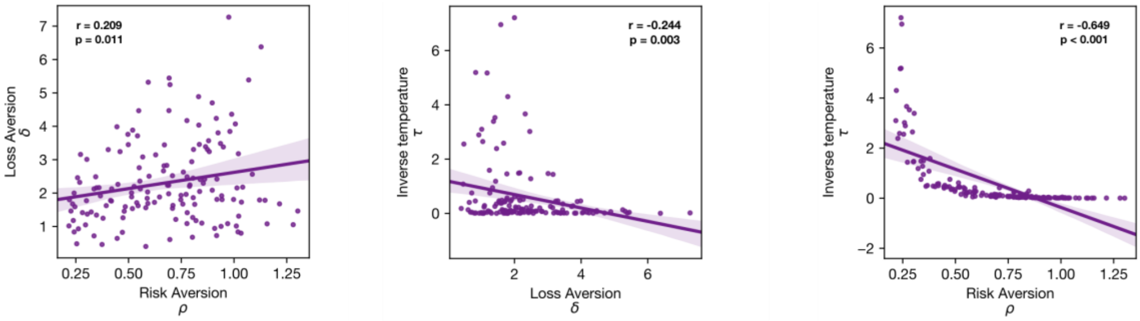

Supplement: Supplementary File 3. — Within-Model Parameter Correlations. [file cpsy-8-1-102-s3.pdf]
