# Supplementary material for: Reward Sensitivity and Noise Contribute to Negative Affective Bias: A Learning Signal Detection Theory Approach in Decision-Making
Source: Comput Psychiatr. 2024 May 9;8(1):70–84. doi: 10.5334/cpsy.102 (PMC11104415; doi:10.5334/cpsy.102)
Supplement: Supplementary File 4. — Regression Model Sensitivity Analyses. [file cpsy-8-1-102-s4.pdf]

Supplementary File 4: Regression Model Sensitivity Analyses

Our negative affective bias task design collects many binary measurements from a single participant. This means that the computed average is a ratio of binary responses. We chose to model this count proportion (p(mid as high)) with a logistic regression, instead of a linear regression, as linear models may predict ratios less than 0 or greater than 1 which are impossible on our task. Secondly, the log transformation used in the logistic regression means the regression values relate directly to the log odds whereas this would not be the case for the linear model.

We tested the robustness of our approach by comparing our logistic regression modelling the averaged p(mid as high) parameter per participant to a model modelling the individual responses of each participant. The estimates should be the same, as the average is the maximum likelihood estimate of the latent probability of p mid as high (Bishop, 2006, p.70). Further, we compared our estimates to a frequency weighted binomial GLM model, with p(mid as high) weighted by number of responded to horizontal trials (which was different for some individuals as they missed trials, max 40). When comparing results of the models, we see that identical trends emerge, with coefficients very similar, regardless of method chosen. Further, we replicate the significant association between p(mid as high) and reward sensitivity, and the significant association between p(mid as high) and setting noise. Thus, when running the suggested models, they do not affect our overall inference.

logistic regression coefficient	averaged logistic regression model	individual response logistic regression model	frequency weighted binomial GLM
intercept	0.315	0.305	0.306
learning rate	-0.017	-0.021	-0.021
reward sensitivity	0.131	0.138	0.139
setting noise	-0.187	-0.189	-0.189
bias	0.100	0.114	0.114
inverse temperature	0.122	0.125	0.125
risk aversion	0.147	0.149	0.149
loss aversion	-0.035	-0.050	-0.050
age	-0.022	-0.036	-0.036
sex	-0.025	-0.012	-0.012

Bishop, C.M. (2006). *Pattern Recognition and Machine Learning (Information Science and Statistics)*. Springer-Verlag.
